# Developmental Origins of Chronic Renal Disease: An Integrative Hypothesis

**DOI:** 10.1155/2013/346067

**Published:** 2013-08-29

**Authors:** F. Boubred, M. Saint-Faust, C. Buffat, I. Ligi, I. Grandvuillemin, U. Simeoni

**Affiliations:** ^1^Department of Neonatology, Assistance Publique-Hôpitaux de Marseille, Marseille, France; ^2^Aix-Marseille Université, Marseille, France; ^3^Aix-Marseille Université, UMR-S 1076 INSERM, Marseille, France

## Abstract

Cardiovascular diseases are one of the leading causes of mortality. Hypertension (HT) is one of the principal risk factors associated with death. Chronic kidney disease (CKD), which is probably underestimated, increases the risk and the severity of adverse cardiovascular events. It is now recognized that low birth weight is a risk factor for these diseases, and this relationship is amplified by a rapid catch-up growth or overfeeding during infancy or childhood. The pathophysiological and molecular mechanisms involved in the “early programming” of CKD are multiple and partially understood. It has been proposed that the developmental programming of arterial hypertension and chronic kidney disease is related to a reduced nephron endowment. However, this mechanism is still discussed. This review discusses the complex relationship between birth weight and nephron endowment and how early growth and nutrition influence long term HT and CKD. We hypothesize that fetal environment reduces moderately the nephron number which appears insufficient by itself to induce long term diseases. Reduced nephron number constitutes a “factor of vulnerability” when additional factors, in particular a rapid postnatal growth or overfeeding, promote the early onset of diseases through a complex combination of various pathophysiological pathways.

## 1. Introduction

Cardiovascular diseases ((CVD) hypertension, coronary disease and stroke, and heart failure) are one of the leading causes of mortality in industrialized countries, and the prevalence is increasing in emerging societies. All cardiovascular diseases account for 4.3 million deaths per year in the European Union, and the prevalence of chronic heart failure in the United States of America is approximately 6 million [1, 2]. In industrialized countries, hypertension (HT) affects 25% to 35% of the global population and reaches 60% to 70% of the population aged 60 or more. Hypertension is the principal risk factor of death worldwide [3]. It increases the severity of ischemic vascular diseases and, with obesity and type 2 diabetes, is one of the important risk factors for chronic kidney disease (CKD). Chronic kidney disease is defined as reduced glomerular filtration rate (GFR) up to end-stage renal disease (ESRD), proteinuria, or both. Prevalence of ESRD, estimated to be 0.5–2.5‰ worldwide, is increasing in several countries [4]. In turn, impaired renal factor favors the development of and amplifies the severity of CVD [5–7]. 

During the last two decades, it has been raised the concept of developmental programming of adult chronic diseases (Developmental Origins of Health and Disease (DOHaD)) [8, 9]. The pathophysiological and molecular mechanisms involved in the early programming of CKD are multiple and partially understood. Reduced nephron endowment has been proposed as playing a determinant role [10–13]. Reduced nephron number is responsible for an adaptive single nephron glomerular hyperfiltration. The consecutive glomerular hypertension may lead over a long time to renal injury, proteinuria, impaired GFR, and hypertension [14]. However, this mechanism is still discussed, and recent experimental studies have failed to show such a link [15–21]. 

This review discusses factors which influence nephron endowment and the complex relationship between nephron endowment and chronic kidney disease. We hypothesize that the developmental “programming” of chronic kidney disease is a complex phenomenon. It may integrate different factors and pathophysiological pathways. Reduced nephron number constitutes a “factor of vulnerability” which is insufficient by itself when it is moderate. In such a situation, early onset of CKD occurs with additional factors including early growth and nutrition. 

## 2. Developmental Origins of CKD

This concept states that chronic and noncommunicable diseases that are currently observed at adulthood have origins in the fetal and perinatal periods of life. Events or stimulus during particular stages of development can alter permanently the structure and function of various systems. After a silent period, diseases occur at adulthood. David Barker and colleagues discovered in the 1980s', in a cohort of people born in Hertfordshire, UK, at the beginning of the nineteenth century, that the mortality ratio due to coronary heart disease was inversely correlated with birth weight [22, 23]. Low birth weight ((LBW), birth weight ≤ 2500 g) was associated with increased rate of mortality. A number of subsequent epidemiologic and experimental studies confirmed this association and the association with other chronic diseases including hypertension, obesity, insulin-resistance and type 2 diabetes [11, 12, 24–29]. It is of note that low birth weight can be related to either intrauterine growth restriction (IUGR) or preterm birth. Recently, other perinatal factors including maternal obesity, maternal diabetes, fetal exposure to specific drugs and preterm birth have been reported to alter the development of various systems increasing the risk for long term diseases [30, 31].

### 2.1. Birth Weight and Chronic Kidney Disease

More recently the risk of chronic kidney disease (CKD) has been related to low birth weight [32–35]. In a population-based study, the estimated glomerular filtration rate (eGFR) has been shown to increase of 2.6 to 7 mL/min per each kilogram increase in birth weight [33, 36]. In a case control study, Lackland et al. have shown in a population of South Carolina, USA, that the odds ratio for end-stage renal disease (ESDR) was 1.4 (95% confidence interval, 1.1–1.8) in adults with birth weight below 2.5 kg [32]. Such results have been recently confirmed in a Norwegian study (the Medical Birth Registry and the Norwegian Renal Registry) where patients with birth weight < 10th percentile had a relative risk (RR) for ESRD of 1.7 (95% confidence interval 1.4 to 2.2) [35]. Finally, LBW is associated with a more rapid progression of various kidney diseases such as membranous and IgA nephropathies, nephrotic syndrome, renal cystic diseases, or kidney disease related to obesity and metabolic disorders [37–41]. In animals, maternal diabetes, maternal obesity, and fetal exposure to drugs can alter nephrogenesis and impair renal function on the long term. Such effects have to be demonstrated in humans.

Adults born preterm constitute an emerging population at risk for cardiovascular and renal diseases. Prenatal and postnatal events may influence renal function and structure later on. The Dutch POP study revealed an inverse relationship between birth weight and long term urinary microalbumin/creatinine ratio and plasma creatinin level in young adults born preterm [42]. Increased microalbuminuria and decreased glomerular filtration rate were observed in patients who were born small for gestational age (SGA) [43]. Impaired renal function has been reported in preterm children with previous history of neonatal hypotension and renal dysfunction [44, 45]. Data are scarce regarding renal structure [46, 47]. Recently, Hodgin et al. have reported 6 adults born preterm (mean age of 32 years) with isolated proteinuria and focal segmental glomerular sclerosis [47]. Aside from renal consequences, preterm birth is associated with early markers of cardiovascular disease and higher risk of HT [48–51]. Preterm birth has to be taken into account as a risk factor of CKD since approximately 130 million infants are born preterm worldwide (frequencies vary from 5.5% to more than 12%) and the prevalence is increasing [52]. Moreover, with a significant improvement in perinatal care, the number of preterm infants reaching adulthood is increasing. 

### 2.2. Early Postnatal Growth and the Risk of CKD

While a rapid postnatal growth during childhood and infancy favours the development of cardiovascular diseases, obesity, and type 2 diabetes; the consequences on renal function and structure are relatively unknown in humans [53–57]. The critical period at which the organism is more sensitive to nutrition and growth is still being debated. Faster weight gain during the first 6 months of life, promoted by a high protein diet, favours the accumulation of the metabolic visceral adipose tissue, reduces insulin sensitivity, and increases blood pressure later in life [56–58]. These effects are exacerbated in low birth weight infants related to preterm birth, IUGR, or both [53, 54, 59–62]. In a longitudinal study of a Finnish cohort, Barker et al. showed that adults who developed coronary heart disease or hypertension were born small, grew slowly within the first months of life, and caught up the BMI early in infancy [53–59]. The proportion of hypertension was higher in patients who were born with LBW and were overweight at adulthood. In contrast, breastfeeding and/or slow postnatal growth appear as a protective factor in LBW infants [63, 64]. Indeed, breastfeeding prevents on the long term the development of central adiposity and obesity, a major risk factor of metabolic and cardiovascular diseases [65, 66].

Similar effects have been reproduced in animals. We, and others, have shown that early postnatal overfeeding, obtained by reduction of litter size and limited to the suckling period, induces obesity, cardiovascular, metabolic, and renal diseases in ageing adult rat offspring [67–70]. Such effects were amplified in IUGR offspring [16, 71, 72]. Blood pressure, fasting insulin, and leptin levels are elevated in young adults IUGR rat offspring nourished during the peripubertal period by a hypercaloric diet (applied after the weaning) [72]. The underlying mechanism is complex. Early overfeeding/overgrowth is associated with overactivity of the sympathetic nervous activity, upregulation of the HPA-axis, early hyperinsulinism, and hyperleptinemia. Hyperinsulinism affects endothelial nitric oxide synthase (eNOS), and hyperleptinemia stimulates the sympathetic nervous system activity. Sustained alteration of the control of appetite with leptin resistance and hyperphagia may exacerbate such metabolic, hormonal, and vascular disorders and hence favour the development of cardiovascular and renal diseases [68, 73]. In contrast, adult diseases can be prevented by slow postnatal growth and manipulation of diet early during the development. In rodents, an increase in litter size (a model of neonatal undernutrition) or prolonged maternal gestational low protein diet after birth and during the neonatal period prevents, in normal birth weight and in IUGR offspring, metabolic disorders and adiposity, long term hypertension and glomerular sclerosis [11, 74–77]. In the same way, we have observed that renal function and structure were unaffected in ageing IUGR offspring with a slow postnatal growth [16]. Such a considerable influence of early growth and nutrition on long term blood pressure in rats has been observed in other species, especially in sheep [78, 79]. Finally, adult hypertension and salt sensitive hypertension (52-week-old animals) could be prevented by placing IUGR rat offspring on a low salt diet just 3 weeks after weaning [80]. 

Altogether, these findings show that early postnatal nutrition (protein/caloric diet, sodium intakes…) and early postnatal growth exert a considerable influence on adult health. Early growth and nutrition can modulate the “fetal programmed” adult chronic diseases. While a rapid postnatal growth and/or overfeeding enhances the “vulnerability state” acquired *in utero* and accelerates the development of adult diseases (“mismatch hypothesis”), a slow postnatal growth and breastfeeding in particular (possibly through reduced protein and sodium intakes) tend to prevent such diseases. 

## 3. Birth Weight, Nephron Endowment and CKD

### 3.1. Nephron Number and CKD

It has been proposed, for a long time, that the pathogenesis of hypertension and chronic kidney disease involves a reduction of nephron number [14, 81–84]. According to the scheme proposed by Brenner et al. (based on clinical data and experimental studies), a decrease in the filtration surface area due to reduced nephron number is associated with an adaptive increase in single nephron glomerular filtration rate (SNGFR). Nephrons undergo structural changes with glomerular and tubular enlargement responsible for renal hypertrophy. Glomerular capillaries enlargement affects podocyte physiology and increases glomerular hypertension through exacerbating the transmission of systemic blood pressure into enlarged glomerulus. In parallel, other physiological changes occur including salt retention, higher volume strokes and cardiac output, resetting in pressure-natriuresis mechanisms and elevated peripheral vascular resistance. They contribute to elevate blood pressure levels [14]. Over a long time a vicious circle takes place responsible for glomerular sclerosis, impaired GFR, and systemic hypertension. The hemodynamic adaptive mechanism is accompanied by molecular and biomolecular changes including inflammation, upregulation of the renin angiotensin system (RAS), and the production of nitric oxide and of reactive species which participate to renal injury [85]. Such a renal mechanism has been proposed as a pathophysiological mechanism linking low birth to long term hypertension and chronic kidney disease [11–13]. 

However, reduced nephron number is not systematically associated with hypertension and impaired GFR, especially when it is moderate. In humans, Hughson et al. did not find this relationship in a group of African-American adults [86]. Several experimental studies failed to demonstrate hypertension and glomerular sclerosis after renal mass resection or congenitally reduced nephron endowment [15–21]. We have recently shown that blood pressure and glomerular sclerosis were unchanged in 22-month-old IUGR ageing males and females rat offsprings with a significant reduction of nephron number (by an average of 25% to 30%) [16]. All these findings suggest that the relationship of reduced nephron number with hypertension and chronic kidney disease is, in fact, more complex and involves various factors. It depends, in part, on the severity of nephron number deficit, the degree of the single nephron glomerular hyperfiltration, or both.

### 3.2. Birth Weight and Nephron Endowment

The development of the kidney is a complex process in mammalian. The time at which nephrogenesis ends differ according to species: in rodents, nephrogenesis continues after birth up to postnatal days 7 to 10, whereas in sheep, the nephrogenesis is achieved before birth at gestational days 125–130 (the normal duration of gestation is 145–150 days). In human, the nephrogenesis is completed by 34–36th weeks of gestation, that is, before birth. About 60% of the nephrons develop during the third trimester of gestation. The definitive kidney, the metanephros, develops from the specific interaction between the epithelial ureteric bud (UB) and the undifferentiated metanephric mesenchyme (MM). An insignificant event occurring during the early stage of nephrogenesis (the branching morphogenesis) can have dramatic effects on the final nephron number (nephron endowment). 

#### 3.2.1. Nephron Number in Human

Nephron number varies widely in the general population and ranges from 2 to more than 10-fold [87–92]. In the Monash series, which included 420 kidneys obtained at autopsy from adults and children from different populations (Aboriginal Australians and white Australians, Senegalese, and white Americans and African Americans), the mean nephron number per kidney was around 900,000 ranging 13-fold from about 210,000 to more than 2,000 000 [88, 90, 91]. Such variability may be explained by genetic and environmental factors, or both. Aboriginal Australians have lower nephron number. Alterations in specific DNA sequences are associated with renal agenesis and hypoplasia [93, 94], and few genetic polymorphisms have been associated to changes in renal volume (a surrogate of nephron mass) [95–97]. 

The principal factor which determines nephron number is birth weight [90], but it is not the only one. Nephron number can vary 3-fold when birth weight is situated within normal range, that is, 3000 g–3500 g [90]. Low birth weight is associated with reduced nephron number. Intrauterine growth restriction ((IUGR) birth weight <10th percentile for gestational age) decreases the nephron number by an average of 30–35%, whereas the effects of preterm birth are still unknown [87, 98, 99]. In preterm infant, the nephrogenesis has to continue in a potentially unfavourable environment. Reduced kidney size and volume have been reported in children and young adults born preterm and in the ones who had postnatal growth restriction [100–103]. Data from autopsy studies which included kidneys from preterm infants who died during the neonatal period at different gestational and postnatal ages showed signs of accelerated maturation of nephrogenesis with enlarged glomeruli [104, 105]. Similar glomerular hypertrophy was observed in premature baboon (E125/E185), with however a preserved nephron endowment [106]. These findings suggest that postnatal nephrogenesis is altered in sick, preterm infants. Babies included in these studies were likely to be the sickest among the patients and to have suffered a prolonged postnatal “stress” which might compromise the postnatal nephrogenesis. The formation of additional nephrons could be preserved in a part of preterm infants who are less immature and have uncomplicated neonatal care or optimal neonatal growth. More studies are clearly needed to assess factors influencing the postnatal renal development in preterm infants. 

#### 3.2.2. Lessons from Experimental Studies

**Figure 1:**
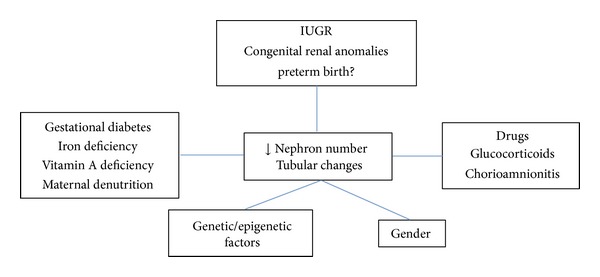
Factors influencing nephron endowment.

Various factors can alter nephrogenesis [12, 87, 99, 107] (Figure 1). Maternal low protein diet, vitamin A deficiency and maternal iron deficiency, uterine arteries ligation, maternal gestational administration of glucocorticoids, or other drugs (antibiotics) lead in most cases to IUGR and to a reduced nephron number by an average of 20%–50%. Maternal gestational diabetes in rodents can alter fetal nephrogenesis as well [12, 99, 108, 109]. In sheep, chorioamnionitis induced by intra-amniotic injection of lipopolysaccharides (LPS) at embryonic days E121 reduced fetal nephron number by 20% [110]. We showed previously in 20-day-old rat foetuses that maternal low protein diet (MLP) reduced permanently the nephron number by an average of 30% [16, 111]. 

The underlying pathophysiological mechanism is incompletely known. Reduction of nephron number may result from an imbalance between pro- and antiapoptotic factors towards apoptosis. Downregulation of the renal renin angiotensin system, fetal overexposure to glucocorticoids, or altered midkine expression has been reported in various IUGR models [99, 112–118]. The expression of specific genes involved in nephrogenesis is altered (Pax2, GDNF) [113, 115]. We found that expression of around 20% of the genome is altered in the fetal kidney of IUGR rat offspring exposed *in utero* to maternal low protein diet [111, 119]. The expression of genes involved in cell maintenance and signal transduction was decreased, and those belonging to the vascular prothrombotic pathway and to the complement components were considerably overexpressed [111, 119]. The effects of fetal environment on nephron endowment may be epigenetically mediated [119–121]. Hypomethylation of the gene p53 has been associated with reduced nephron number in a rat model of placental insufficiency [121]. In addition, we showed in the kidneys of IUGR fetal offspring changes in the expression of genes coding for specific enzymes involved in epigenetic machinery [119]. Changes in epigenetic marks could be transmitted to the next generation and be responsible for “transmitted” nephron deficit. In rat, offspring (second generation, F2) from parents exposed prenatally to maternal gestational low protein diet (first generation, F1) had normal birth weight but 30% to 40% reduction in nephron number [120]. Additional studies are however needed to confirm and to eventually explain such a transgenerational transmission of acquired phenotype.

Birth weight is not the only predictive factor of the nephron endowment. In animal, the nephron endowment is also characterized by a certain rate of variability. In rodent, despite strictly controlled conditions, the nephron endowment can vary by an average of 10% to 15% for a birth weight situated within normal range [122, 123]. Events that occur during the early stage of nephrogenesis can induce a nephron deficit without affecting birth weight [124–127]. Exposure to maternal low protein diet and administration of a short course of glucocorticoids during the early stage of nephrogenesis, in rodents (E14–17) and in sheep (E80), are sufficient to reduce nephron endowment (−20% to −40%) without inducing low birth weight [124–126]. Interestingly, the nephron endowment can be preserved in IUGR offspring, especially when IUGR is spontaneous or when it occurs late in gestation [123, 128]. In summary, the more the process of IUGR appears early in the gestation, the more the nephrogenesis is affected, and the nephron endowment severely reduced. The early stage of nephrogenesis constitutes a “critical window” when an event can alter profoundly and durably the nephrogenesis. 

Postnatal environment can influence nephron endowment in certain situations when the nephrogenesis continues after birth. Recent studies in rodents showed that neonatal undernutrition (−30% to −40%, through increasing litter size) reduced nephron endowment (−20%), but early neonatal overfeeding (through reduction of litter size) enhanced postnatal nephrogenesis (+25%) [70, 129]. However, this last effect was not observed in IUGR offspring (induced by maternal low protein diet). Indeed, while pups displayed a rapid catch-up growth within the first 15 days after birth, the nephron endowment failed to be restored. Interestingly, Wlodek et al. found a relative restoration of nephron endowment when IUGR pups were switch to normal lactating dams [130]. In the last study, IUGR was induced by uterine ligation at embryonic day 17. This discrepancy may result from a marked deficit in nephron precursors observed in fetus exposed to maternal low protein diet at the early stage of fetal development [115, 116]. 

Such findings emphasize that birth weight is a predictive factor of nephron endowment but is certainly not the only one. Nephron endowment may result from a complex process which integrates the interaction of the fetal environment (or postnatal environment in preterm infants) and the genetic background. Normal birth weight does not always signify a sufficient nephron endowment and low birth weight a severe nephron deficit. The relationship between birth weight and nephron endowment is not so linear and it could be difficult to predict nephron endowment for an individual based only on birth weight. 

## 4. Birth Weight and Chronic Kidney Disease: An “Integrative” Hypothesis

**Figure 2:**
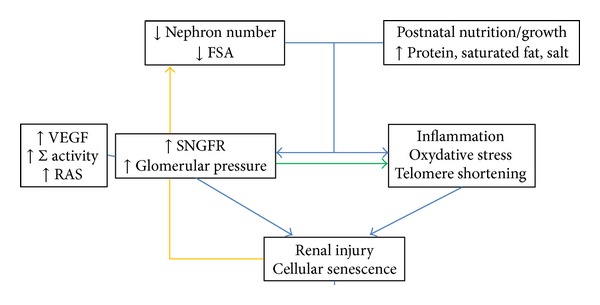
Proposed pathophysiological mechanism of renal injury (glomerular endothelium barrier disruption, podocyte dysfunction, interstitial fibrosis, tubular ischemia). FSA: filtration surface area; GFR: glomerular filtration rate; SNGFR: single nephron glomerular filtration rate; RAS: rennin angiotensin system; VEGF: vascular endothelial growth factor system; ∑: sympathetic nervous system.

**Figure 3:**
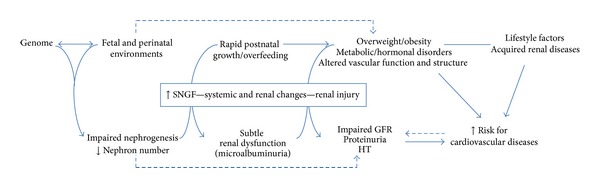
Developmental origin of hypertension and chronic kidney disease: an “integrative hypothesis”.

Relationship of birth weight with chronic kidney disease is complex and integrates various factors and pathophysiological mechanisms of which nephron number plays a pivotal role (Figures 2 and 3).

Predominant factor which determines nephron endowment is birth weight. However, it is not the only one. Nephron endowment, acquired at birth (or after birth for preterm infants), may result from an interaction between genetic background and environmental factors. Environment may alter nephrogenesis through epigenetic pathway, and genetic background may make the kidney less or more sensitive to environmental factors. For example, some mice strains are less sensitive to nephron deficit and glomerular sclerosis induced by maternal gestational administration of aminoglycosides [131]. When nephrogenesis is severely impaired, that is, when the nephron deficit is marked, the risk of early impaired renal function and hypertension is elevated. It is the case of infants born with congenital anomaly of kidney and urinary tract. In most cases, alterations in nephrogenesis are subtle and are probably not responsible by themselves to chronic kidney disease. However, such changes constitute a “factor of vulnerability.”

Various postnatal factors can induce a single nephron glomerular hyperfiltration or glomerular hypertension and together with a reduced filtration surface area may accelerate the occurrence of CKD. Nutrition or growth early in life is one of them. We and others have shown that a rapid postnatal growth and overfeeding (high caloric and protein intakes) early in life induced in young adult IUGR rat offspring a renal hypertrophy and proteinuria (a surrogate of a glomerular hyperfiltration or glomerular endothelium barrier injury) [12, 16, 70, 117, 132]. Protein diet may play an important role since it is known for a long time that high protein intakes in adult animals induce glomerular hyperfiltration, renal hypertrophy, and long term glomerular sclerosis [74]. In another study, twelve-week-old IUGR rat offspring exposed postnatally to a high protein diet (+30%) displayed glomerular hypertrophy, podocyte damage, and early signs of interstitial fibrosis [133]. On the other hand, a slow postnatal growth prevents the development of renal disease in IUGR and normal birth weight offspring. The renal effects of a high protein diet are more marked when the kidney is immature due to its higher capacity than the adults' to adapt renal hemodynamic (with higher sensitivity to the RAS) [134]. This adaptative mechanism is associated with various changes including upregulation of the renal RAS of the VEGF system and overactivity of the sympathetic nervous system. Inflammation and oxidative stress have been demonstrated in these kidneys as well (Figure 2) [135–139]. These changes may initiate a “renal stress,” an infraclinical renal injury. Indeed, kidney of IUGR overfed offspring which displayed a rapid postnatal catch-up growth expressed stress-induced senescence protein markers (p16, p21) and telomere shortening [135–140]. Telomere shortening, related with “oxidative stress,” favours premature cell death (Figure 2). However, it is unknown whether such changes persist on the long term and whether it can be reversed.

Some of these experimental findings have been reported in human. Early high protein diet and rapid growth rate tend to induce a renal hypertrophy. Two recent studies have prospectively evaluated the effects of different diets on renal structure (using ultrasound) in infants born at term with birth weight adapted for gestational age [141, 142]. In the first study, when a group of formula-fed infants was compared to breastfed infants, the authors showed a 25% increase in renal volume at 3 months of age. This effect was transient and was no longer observed at 18 months when all infants were on mixed diet [141]. The second study aimed to investigate the renal effects of two low (1.25 g/dL, average breastfeeding) and high protein (2.05 g/dL, +60%) formula diets in healthy infants [142]. At 6 months of age, while no differences were found between breastfed and low protein formula-fed infants, the kidney volume (and the relative volume of the kidney/body surface area ratio) was 10% higher in high protein formula-fed infants. This renal effect may result from a single nephron glomerular hyperfiltration induced by high protein intakes as demonstrated in experimental studies. 

Early nutrition and growth can alter renal function and structure through other pathways. In animal, early postnatal overfeeding is associated with hypertension, obesity, and type 2 diabetes, known as risk factor for CKD. A rapid postnatal catch-up growth and/or overfeeding is associated with hyperleptinemia, hyperinsulinism and insulin-resistance, upregulation of the RAS and HPA-axis, and overactivity of the nervous sympathetic activity (see above). Such effects are responsible for impaired endothelium-dependent vasodilatation, systemic vasoconstriction, oxidative stress, and systemic inflammation which alter in turn vascular structure and arterial stiffness and lead to hypertension. Obesity and hyperglycaemia induce a single nephron glomerular hyperfiltration [143, 144], but it is still unknown if such changes are sufficient by themselves to affect renal structure. Experimentally, hyperglycaemia (administration of streptozocin ± insulin therapy, equivalent of type 1 diabetes) in young adult IUGR offspring induces single nephron glomerular hyperfiltration and proteinuria but does not affect the glomerular structure on the long term (10 mo) [145, 146]. These findings may be explained by the unchanged blood pressure and the associated weight loss which have limited the adverse renal effects of hyperglycaemia. Indeed, blood pressure plays an important role. Comparing two models of obesity-induced renal injury, do Carmo et al. demonstrated the detrimental role of hypertension on renal structure [147]. Finally, in rodents, early overgrowth/overfeeding alters the central control of appetite with sustained hyperphagia. This last effect can have detrimental effects on the kidney. In industrialized countries, a large part of the population is exposed to hypercaloric diet named “western diet.” This diet, characterized by high carbohydrates, salt, protein, and saturated fat contents, is known to increase the risk of atherosclerosis and cardiovascular disease and to promote the development of glomerular sclerosis [148–150]. These effects are mediated by the overactivity of the sympathetic nervous system, inflammation, and oxidative stress (exacerbated in part by angiotensin II) [149]. In human, high protein diet accelerates the deterioration of GFR in adults with low GFR [151]. Other nutrient and are of importance. High salt intakes are known to increase blood pressure levels and the risk of CKD, especially in overweight patients [80, 152, 153]. Such a salt sensitivity may be enhanced in IUGR offspring. In IUGR rat offspring, reduced nephron number is associated with tubular changes including increased expression of the renal tubular Na^+^ : K^+^ : 2Cl^−^ cotransporter (NKCC2) and altered the Na^+^ : K^+^ ATPase activity responsible for a tendency to sodium retention and a higher sensitivity to high salt intake [80, 154–160]. One can easily understand that this nutritional factor may amplify the vascular and systemic effects of pre-existing type 2 diabetes, obesity, and hypertension and favour the development of CKD. 

Altogether these additional factors in combination with “vulnerable” kidneys accelerate the onset of CKD through the increase in the SNGFR, the reinforcing of the exacerbation of the pre-existing “renal stress”, and through the transmission of elevated systemic blood pressure to enlarged glomerular capillaries. Hence, the kidney appears both as the underlying pathophysiological mechanism and as the target organ of developmental programming of CKD.

## 5. Implications for Followup, Nutrition, and Prevention in Patients at Risk

Early life conditions are of particular importance in the comprehension of chronic kidney disease (CKD). Their importance equals or may exceed that of other later environmental risk factors. Various systems, including the kidney, are permanently altered. Reduced nephron number, with renal tubular changes, constitutes a “factor of vulnerability” when additional factors as the early postnatal growth/nutrition promote early onset of hypertension and CKD through various pathways. Despite this clear pathophysiologic rationale, a number of points still need to be addressed to allow the design of effective preventive strategies. The criteria for a subject being considered at particular risk need to be defined. This is the less easy since a number of epidemiologic and clinical studies show that the long term programming of hypertension and of renal disease does not occur only in well-defined, pathological conditions such as low birth weight, preterm birth, and exposure to maternal diabetes in pregnancy. Even in apparently healthy children, estimated glomerular function has been shown to be correlated with size at birth [161]. Questions such as the optimal nutrition of low birth weight infants, whether due to intrauterine growth restriction, preterm birth or both, the optimal followup of vascular, metabolic, and renal functions, and possible nutritional and pharmacological interventions remain unanswered. Postnatal undergrowth/undernutrition is associated with impaired neurological function and potentially death in certain regions of the world [162]. Future research may aim to clarify early biomarkers and markers of nephron endowment and early renal injury in order to determine optimal perinatal nutrition and the eventual prophylactic measures to be applied to infants at increased risk of developmentally programmed adult diseases. However, simple preventive measures such as promoting breastfeeding (at least 6 mo) and physical activity early in childhood and establishing early program of nutritional education and public nutritional policies (reduced sodium, carbohydrates and saturated fat in ready meals, e.g.) are now feasible and can have significant impact on public health (as suggested by experimental studies).
